# Supplementary material for: A New Topical Candidate in Acne Treatment: Characterization of the Meclozine Hydrochloride as an Anti-Inflammatory Compound from In Vitro to a Preliminary Clinical Study
Source: Biomedicines. 2022 Apr 19;10(5):931. doi: 10.3390/biomedicines10050931 (PMC9138413; doi:10.3390/biomedicines10050931)
Supplement: Supplementary file 1 [file biomedicines-10-00931-s001.zip › biomedicines-1668422-supplementary.pdf]

SUPPLEMENTAL MATERIALS

Toxicological evaluation of meclozine. Before performing clinical evaluation of meclozine, reglementary toxicological tests were realized under the cosmetic standards requirements. Bacterial reverse mutation assay (Ames test), ocular irritation test (HET CAM), cutaneous irritation test (SkinEthic), skin sensitization tests (KeratinoSens™ and Direct Peptide Reactivity Assay) and phototoxicity test (3T3 NRU) were performed and are available in Supplemental information (IDEA Lab, Martillac, France). These studies concluded that meclozine gel was ocular irritant, not mutagenic, not pro-mutagenic, not skin irritant, not skin sensitizer and not phototoxic. Then, assessment, under dermatological control, of skin compatibility after single application in a 48 consecutive hours semi-occlusive patch-test on 10 volunteers concluded that 1% meclozine gel was non-irritant.

TABLES AND FIGURES

TABLE

Supplementary Table S1: Pre-clinical toxicological assay[a] on meclozine

Toxicity test	Nature	Concentration (µg/ml)	Result
HET-CAM test	Evaluation of occular irritant potential	Pure	Classified as irritant
3T3 NRU	*In vitro* phototoxicity (OCDE 432 guideline)	7.81 - 1000	Classified as not phototoxic
Bacterial reverse mutation assay	Determination of the mutagenic activity (Ames test) (OCDE guideline 471)	19.2 - 1923	Not mutagenic<br>Not pro-mutagenic
Keratinosens	*In vitro* sensitization on keratinocytes	1 - 1000	Classified as not skin sensitizer
Direct Peptide Reactivity Assay (DPRA)	*In chemico* skin sensitization (OCDE 442C guideline)	10000	Negative prediction
Cutaneous irritation	*In vitro* cutaneous irritation on SkinEthic model (OCDE 439 guideline)	Pure	Meclozine must not be classified

[a]Assays performed by the IDEA Compagny

Supplementary Table S2: Cutaneous compatibility test[a] of meclozine

Nb of volunteers included	Sex	Age	In-situ reaction of product	Individual score of product	In situ reaction on Control site 1	In situ reaction on Control site 2
1	F	60	none	0.00	none	none
2	M	62	none	0.00	none	none
3	M	52	none	0.00	none	none
4	F	44	none	0.00	none	none
5	F	65	none	0.00	none	none
6	F	62	none	0.00	none	none
7	F	65	none	0.00	none	none
8	F	62	none	0.00	none	none
9	F	52	none	0.00	none	none
10	F	6	none	0.00	none	none

[a]Assays performed by the IDEA Compagny

Supplementary Table S3: Lesion count in volunteers during the 12-weeks study

Patient number[c]	Treated group[a]		Control group[b]	
	Baseline	Week 12	Baseline	Week 12
	ASI[d]	ASI	ASI	ASI
**1**	19.00	12.00	35.25	19.75
**2**	46.25	35.75	5.50	**5.50**
**3**	15.00	9.50	15.00	**15.00**
**4**	24.00	16.25	16.00	21.00
**5**	25.50	**25.50**[e]	12.75	**12.75**
**6**	13.50	16.00	31.50	**31.50**
**7**	29.00	33.25	18.50	11.00
**8**	14.50	17.00	13.50	14.00
**9**	20.50	14.50	36.75	17.75
**10**	13.50	13.00	42.75	36.50
**11**	17.25	8.25	39.50	39.00
**12**	24.50	24.25	9.50	8.000
**13**	20.25	18.75	64.25	53.75
**14**	38.50	30.00	63.00	45.75
**15**	22.75	36.25	28.50	**28.50**
**16**	56.25	25.50	27.25	24.25
**17**	13.25	**13.25**	44.25	**44.25**
**18**	64.25	48.25	12.00	**12.00**
**19**	18.50	14.50	17.00	7.75
**20**	26.75	**26.75**	9.50	10.50
**21**	55.00	39.75	12.75	8.00
**22**	65.50	**65.5**	8.75	**8.75**
**23**	59.75	43.50	54.00	60.75
**24**	50.75	31.00	14.00	15.50
**25**	17.00	14.75	24.25	21.75
**26**	61.75	42.50	52.50	66.00
**27**	11.25	11.75	8.25	11.50
**28**	56.25	40.00	63.75	50.75
**29**	21.25	11.50	14.25	**14.25**
**30**	17.75	12.25	10.25	7.50

[a]Volunteers treated with 2% meclozine pharmaceutical gel.

[b]Volunteers treated with the vehicle gel.

[c]A total of 60 volunteers were included and randomized into two groups of 30 volunteers.

[d]Michaelson's Acne Severity Index (ASI) was evaluated by counting inflammatory lesions (papules, pustules and nodules with size >= 5 mm) and non-inflammatory lesions (open and closed comedones) and calculated as follow: ASI = (comedones x 0.5) + (papules x 1) + pustules x 2) + (nodules x 3).

[e]Values in bold corresponded to the dropped-out volunteers

Supplementary Table S4: Primer used in this study

Name	Forward	Reverse
IL-1β	TCCTGCGTGTTGAAAGATGATAA	CAAATCGCTTTTCCATCTTCTTC
CXCL8/IL-8	TCTTGGCAGCCTTCCTGATT	TTTCTGTGTTGGCGCAGTGT
TNF-α	GAGCACTGAAAGCATGATCC	CGAGAAGATGATCTGACTGCC
GM-CSF	GGAGCATGTGAATGCCATCCAG	CTGGAGGTCAAACATTTCTGAGAT
GAPDH	GCCACATCGCTCAGACAC	GCCCAATACGACCAAATCC

FIGURE

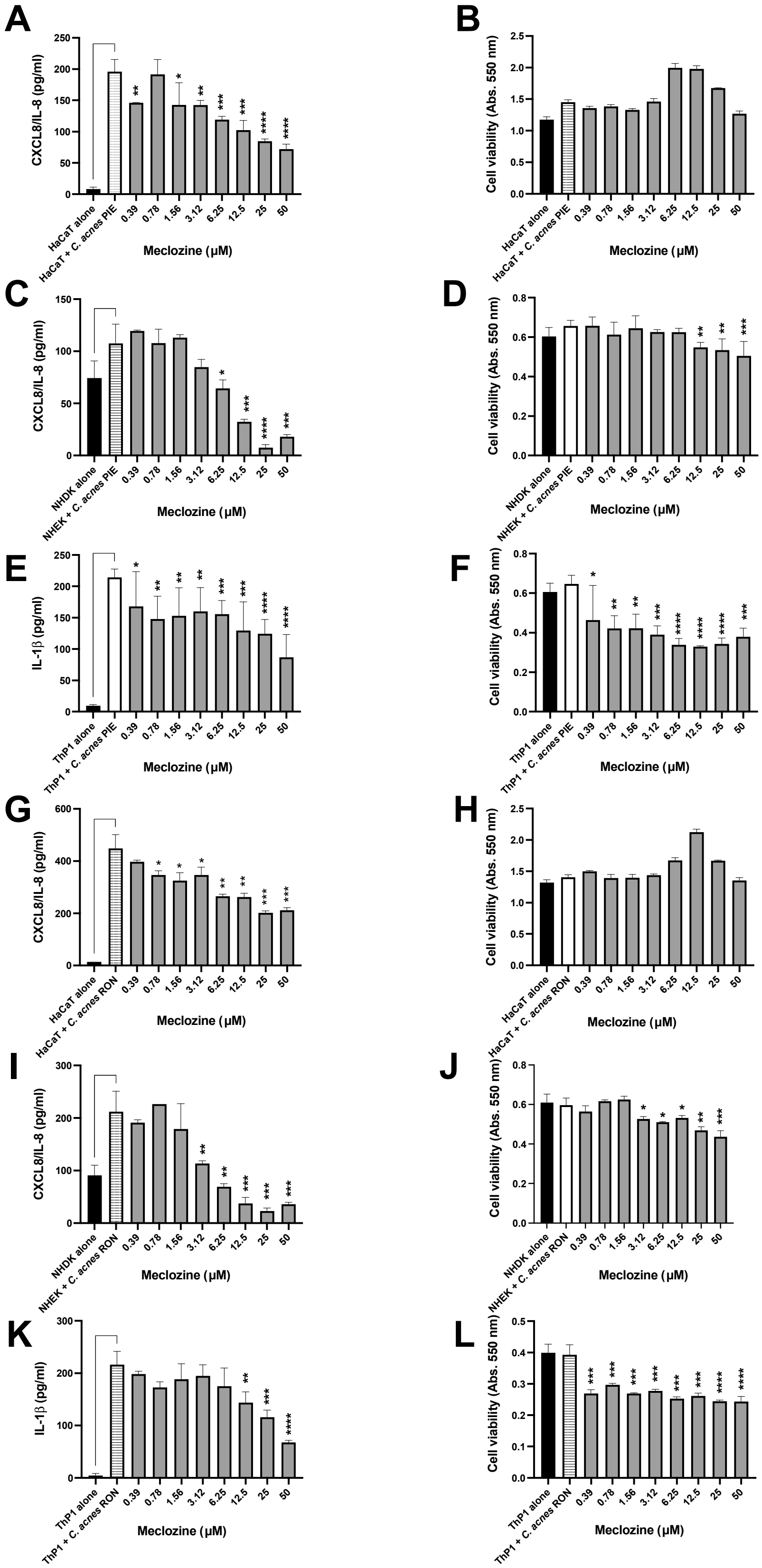
A
CXCL8/IL-8 (pg/ml)
HaCaT alone
HaCaT + C. acnes PIE
0.39
0.78
1.56
3.12
6.25
12.5
25
50
Meclozine (µM)
B
Cell viability (Abs. 550 nm)
C
NHDK alone
NHEK + C. acnes PIE
D
E
IL-1β (pg/ml)
ThP1 alone
ThP1 + C. acnes PIE
F
G
HaCaT + C. acnes RON
H
I
NHEK + C. acnes RON
J
K
ThP1 + C. acnes RON
L

Supplementary Figure S1: Meclozine inhibits the CXCL8/IL-8 and the IL-1β productions in keratinocytes and monocytes stimulated by *C. acnes* PIE and RON strains. (**a**,**b,g,h**) HaCaT cell, (**c,d,I,j**) primary keratinocyte (NHDK), and (**e,f,k,l**) ThP-1 cell were incubated for 24 h with meclozine at concentrations ranging from 0.39 to 50 μM and stimulated with *C. acnes* strain (gray bar). Controls experiments were done with untreated and unstimulated cells (black bar) and with cells stimulated with *C. acnes* only (horizontal line bar). Measurement of CXCL8/IL-8 and IL-1β productions were realized by ELISA and cytotoxicity was determined by the MTT assay as described in Materials and Methods. Data are means ± S.D. of three separate experiments. Statistical significance is indicated by * (P<0.05), ** (P< 0.01), *** (P<0.001), and **** (P < 0.0001), respectively.

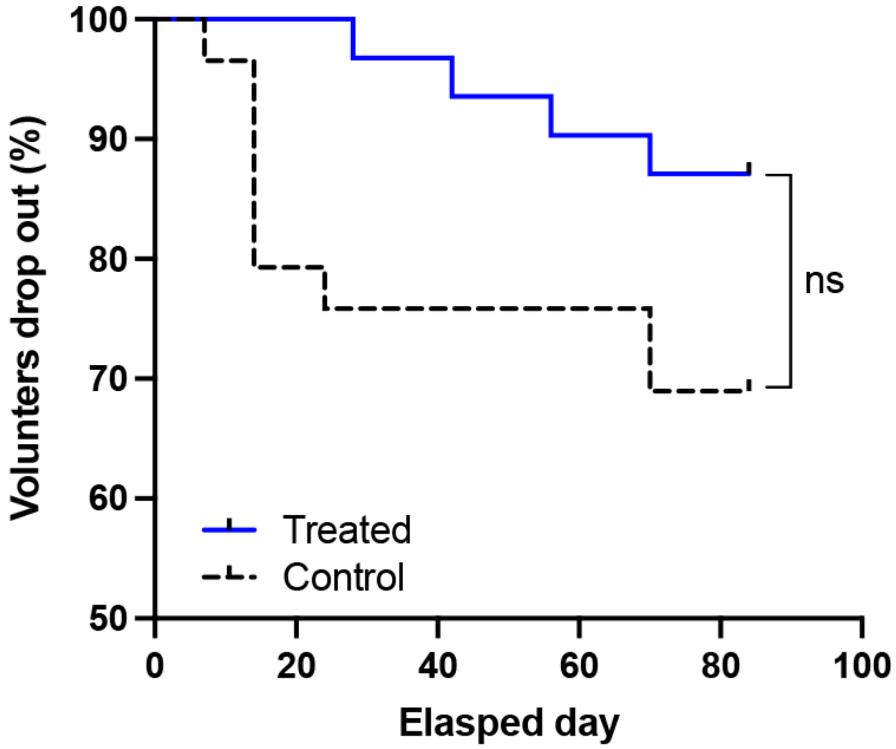

Supplementary Figure S2: Analysis of the drop-out volunteers during the 12-weeks study.

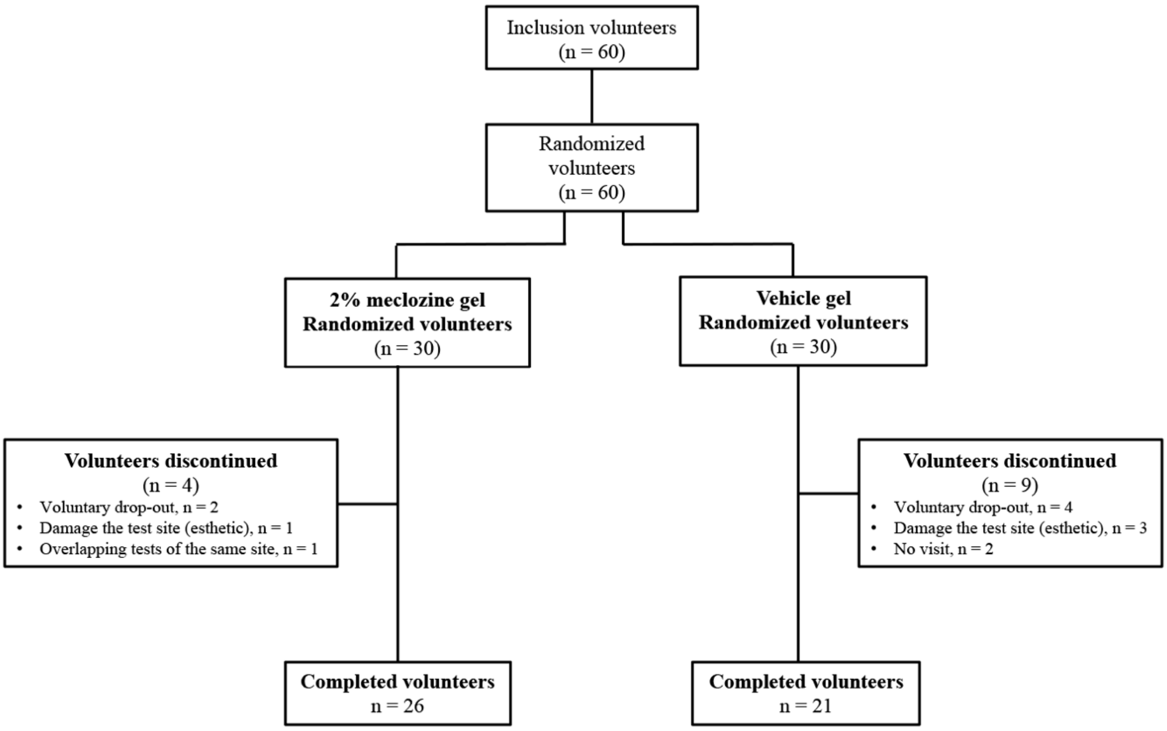

Supplementary Figure S3: Flow chart of study population in the clinical trial.

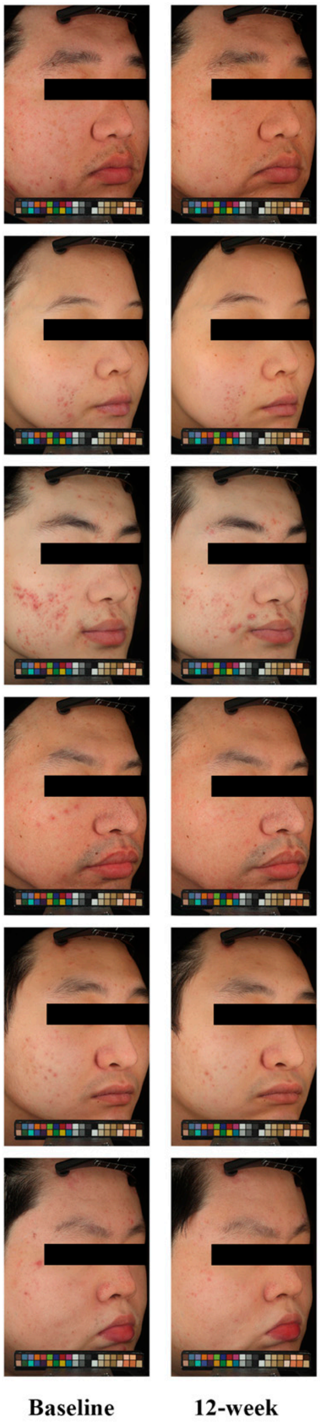

Supplementary Figure S4: Clinical examination of volunteers during the 12-weeks study. Acquisition of facial images was performed by VISIA-CR (Canfield Imaging System, USA) equipped with a forehead clamp enabling fixed angle and distance between the volunteer and the camera at all times. The photography conditions were fixed using the equipped strobe, and Cross-polarized mode was used to take the facial images (left and right lateral).
